# Red blood cell distribution width and carotid intima-media thickness in patients with metabolic syndrome

**DOI:** 10.1186/s12872-017-0481-x

**Published:** 2017-01-28

**Authors:** Dongdong Ren, Juan Wang, Hua Li, Yanyan Li, Zhanzhan Li

**Affiliations:** 1 Emergency Department, Henan Province Hospital of Traditional Chinese Medicine, Zhengzhou, Henan Province 450002 People’s Republic of China; 2 Henan Province Hospital of Traditional Chinese Medicine, Zhengzhou, Henan Province 450002 People’s Republic of China; 3 Department of Oncology, Xiangya Hospital, Central South University, No. 87, Xiangya Road, Changsha, Hunan Province 410008 People’s Republic of China

**Keywords:** Red blood cell distribution width, Carotid artery atherosclerosis, Metabolic syndrome

## Abstract

**Background:**

To evaluate the relationship between red blood cell distribution width (RDW) and carotid intima-media thickness (CIMT) in metabolic syndrome (MetS) patients.

**Methods:**

In this study, we analyzed 803 patients with MetS who underwent carotid ultrasonography examination at Henan Province Hospital of Traditional Chinese Medicine from October 2014 to September 2015. Demographic data were collected using a questionnaire. An automatic biochemistry analyzer measured RDW. Pearson correlation coefficient, multivariate linear and logistic regression was used to evaluate the correlation between RDW and CIMT.

**Results:**

Compared with control group, case group had higher RDW level (*P* < 0.001) and CIMT (*P* < 0.001). CIMT was positively related to RDW (*r* = 0.436, *P* < 0.001). Logistic regression indicated that RDW was a predictor of CIMT ≥ 1 mm. Compared with the first quartile, people with third and fourth quartile level gave obvious higher risk of carotid artery atherosclerotic trend (OR = 1.41, 95% CI:1.01–197; OR = 2.10, 95% CI: 1.30–3.40). Using a cutoff point of 13.9%, RDW predicts elevated CIMT with a sensitivity of 62.1% and a specificity of 70.3%.

**Conclusion:**

High RDW is related to the increased CIMT in MetS patients, which highlights the role of RDW in the progression of elevated CIMT in MetS patients.

## Background

Metabolic syndrome (MetS) is characterized by a cluster of obesity, hypertension, and dyslipidemia-diabetes. It has emerged as a global health issue that affects about 20–30% of adults in many countries [1–3]. Previous studies suggest that MetS is related to adverse cardiovascular outcomes [4]. Carotid artery atherosclerosis is fprogressive disease caused by thickened carotid intima-media thickness (CIMT). It has been suggested that elevated CIMT is associated with coronary artery disease, ischemic stroke, and chronic kidney disease, independent of many known influence factors such as age, body mass index, and smoking [5–7]. The clinical symptoms of thickened CIMT are usually not obvious in MetS patients [8]. Therefore, early identification of increased CIMT is particularly important, which may significantly impact the outcomes.

Red blood cell distribution width (RDW), an index of routine blood examination, reflects the mean corpuscular volume and variability, and is commonly combined with other clinical indices to diagnosis anemia [9]. Lipi and his colleagues reported in 2009 that RDW may be a new inflammatory biomarker [10]. This finding provides a new predictor of potential cardiovascular risks. Recently, many studies suggest that elevated RDW is associated with the increased incidence of cardiovascular events [11–13]. Li et al. found that RDW could predict the early renal function in hypertensive patients with a sensitivity of 76% and a specificity of 70% [14]. In parallel with this, a large cohort study with 3529 consecutive patients indicates high RDW (≥14%) is independently related to higher rates of MetS [15]. Previous study also indicated a positive relationship between RDW and carotid artery atherosclerosis in patients with ischemic stroke [16]. However, there are few studies on the relationship between RDW and CIMT in MetS patients. Since carotid artery atherosclerosis is caused by several inflammatory responses of vascular endothelial injuries, it is inferred that RDW could be involved in the process of carotid artery atherosclerosis. Given the absence of evidence about the association between RDW and CIMT, we think further exploration is needed.

## Methods

### Study population

The present study was based on a cross-sectional design. The study population was selected from our hospital between October 2014 and September 2015. Initially, 915 people were diagnosed with MetS, and those who did not meet the criterion were excluded. Finally, 803 MetS patients were included. The study population was aged from 24 to 54 years old (mean 48.9). More than half of them were males (65.3%). The study was approved by the Ethics Committee of Henan Province Hospital of Traditional Chinese Medicine. All participants were informed about the objectives, and signed the written informed the consents. The whole process would have no privacy disclosure.

### Criterion for inclusion and exclusion

Study population must meet the following criteria: clearly diagnosis of MetS. Patients who had history of cardiovascular diseases (ischemic stroke, coronary heart disease, cerebral infarction, hemorrhagic stroke), or severe renal function impairment, gout, tumor, pregnancy, and severe inflammatory diseases were excluded. Those who have diabetic complications, trauma, malignant neoplasms or operations in the past six months were also excluded.

### Data collection

The general information was collected by a questionnaire. An experienced physician measured height, weight, waist circumference (WC), and hip circumference of each patient. Body mass index (BMI) was calculated by the formulation: one’s weight in kilograms divided by the square of one’s height in meters. The waist to hip ratio (WHR) was also calculated. Blood pressures (SBP: systolic blood pressure, DBP: diastolic blood pressure) were measured three times after 5 min of rest with at least 15 s between measurements with the mean taken as the final BP. If a difference of >5 mmHg was found in any case remeasurement would be done.

Blood for biochemical examinations was drawn from the antecubital vein and examined within 4 h. Blood routine cell counting (red cell, white cell, RDW, platelet, hemoglobin), triglyceride (TG), total cholesterol (TC), high density lipoprotein-cholesterol (HDL-C), low density lipoprotein-cholesterol (LDL-C), and fast plasma glucose (FPG) were measured by an automatic biochemistry analyzer (Beckman Coulter, Inc., Fullerton, CA, USA). Serum creatinine, uric acid, and urea nitrogen were collected by Hitachi Modular System P (Germany). The estimated glomerular filtration rate (eGFR) was calculated by using the formulation [17]: 186 × [SCr]^-1.154^ × [age]^-0.203^× [1.233 If male] × 0.742^20^. The eGFR < 60 mL/min/1.72 m^2^ indicated the occurrence of chronic kidney disease.

An experienced sonographer performed all carotid ultrasonography by using a machine with a frequency of 11 MHz (ATCUM9, USA). We measured the anterior, lateral and posterior longitudinal carotid arteries. Both sides were done to calculate the CIMT. The elevated CIMT was defined as CIMT > 1 mm [18].

### Diagnostic criteria

The diagnosis of MetS was based on the criteria recommended by the Chinese Diabetes Society (CDS) Criterion of MetS [19]. Mets was defined as the presence of three or all of the following items: (1) overweight or obesity: BMI ≥ 25.0 kg/m^2^; (2) hyperglycemia: FPG ≥ 6.1 mmol/L and or 2 h PG ≥ 78 mmol/L, and or diagnosis with mellitus diabetes or drug treatment [20] (3) hypertension: SPB and/or DBP ≥ 140/90 mmHg [21], and or diagnosed with hypertension or drug treatment. (4) TG ≥ 1.7 mmol/L, and or HDL-C < 0.9 mm/L(male) or <1.0 mmol/L(female).

### Statistical analysis

Continuous variables were expressed mean ± standard deviation or median (min-max) depending on whether data conformed to normal distribution (Kolmogorov-Smirnov test). Category variables were expressed as percent. Student’s-t test, Chi-square test or non-parametric test was chosen according to data distribution. The relationships between CIMT and RDW and other variables were evaluated via Pearson or Spearman’s correlation coefficient. Both linear regression and logistic regression were used. Multicollinearity among variables was also assessed by using eigenvalue and condition index before establishment of regression model. All statistical analyses were perfomed on SPSS 18.0 (SPSS inc, USA) with *P* < 0.05 as the statistical significance level.

## Results

### General characteristics of study subject

Finally, 803 MetS patients were included, and divided these patients into a case group with CIMT ≥ 1 mm, and a control group with CIMT < 1 mm. The case group has significantly higher RDW level and CIMT compared with the control group (both *P* < 0.001). The variation coefficient of CIMT is 15.6%. The case group tends to be smoker, and has significantly higher BMI, waist circumference, waist to hip ratio, SBP, triglyceride, LDC-C (all *P* < 0.001), DBP (*P* = 0.007), and total cholesterol (*P* = 0.002). The serum creatinine and uric acid levels in the case group are higher than in control group. Besides, the high-sensitivity C-reactive protein (Hs-CRP), red blood cell and hemoglobin levels are all significantly different between the two groups. No significant difference between groups is found in the rest variables. The general characteristics of the study population are shown in Table 1.

**Table 1:** General characteristics of study population according to the carotid intima-media thickness

Parameters	Control group (*n* = 516)	Case group (*n* = 287)	*P*
Age (year)	48.7 ± 6.9	49.5 ± 5.7	0.051
Sex (male, %)	345 (66.9%)	179 (62.4%)	0.200
Smoking, yes	178 (34.5%)	64 (22.3%)	<0.001
Drinking history, yes	88 (17.1%)	35 (12.2%)	0.067
Hypertension, yes	204 (39.5%)	102 (35.5%)	0.264
Diabetes mellitus, yes	76 (14.5%)	37 (12.9%)	0.473
Body mass index, kg/m^2^	25.3 ± 0.6	25.5 ± 0.7	<0.001
Waist circumference, cm	89.3 ± 8.1	92.4 ± 8.2	<0.001
Waist to hip ratio	0.85 ± 0.1	0.88 ± 0.1	<0.001
Systolic blood pressure, mmHg	143.8 ± 16.9	148.2 ± 18.3	<0.001
Diastolic blood pressure, mmHg	89.0 ± 10.4	90.9 ± 10.5	0.007
Triglyceride, mmol/dL L	1.5 ± 0.5	1.9 ± 0.3	<0.001
HDL-C, mmol/dL	1.1 ± 0.1	1.0 ± 0.2	0.500
LDL-C, mmol/dL	2.7 ± 0.4	3.0 ± 0.5	<0.001
Total cholesterol, mmol/dL	4.7 ± 0.4	5.2 ± 3.4	0.002
Fasting plasma glucose, mmol/dL	6.7 ± 1.65	6.8 ± 2.6	0.278
HbA1c (%)	6.2 ± 1.3	6.4 ± 1.9	0.056
Serum creatinine, mmol/dL	77.8 ± 3.2	79.3 ± 3.3	<0.001
Serum uric acid, μmol/L	323.8 ± 115.2	354.2 ± 125.8	0.003
eGFR, (mL/min/1.72 m^2^)	72.8 ± 35.5	95.5 ± 21.3	<0.001
Blood urea nitrogen, mmol/L	4.7 ± 1.1	4.8 ± 1.1	0.108
Hs-CRP, mg/dL	2.2 ± 0.5	2.4 ± 0.8	<0.001
Red blood cell, ×10^12^/L	4.5 ± 0.4	4.4 ± 0.4	<0.001
White blood cell, ×10^9^/L	6.0 ± 2.4	6.2 ± 2.5	0.135
Red cell distribution width (%)	13.8 ± 0.4	14.3 ± 0.3	<0.001
Blood platelet, ×10^9^/L	215.6 ± 74.2	208.3 ± 54.5	0.056
Hemoglobin,g/L	145.2 ± 12.5	150.1 ± 13.9	<0.001
CIMT (mm)	0.9 ± 0.1	1.2 ± 0.2	<0.001

*HDL-C* high-density lipoprotein cholesterol, *LDL-C* low-density lipoprotein cholesterol, *Hs-CRP* high-sensitive C-reactive protein, *CIMT* carotid intimal-medial thickness

### Correlation analysis

**Fig. 1:**
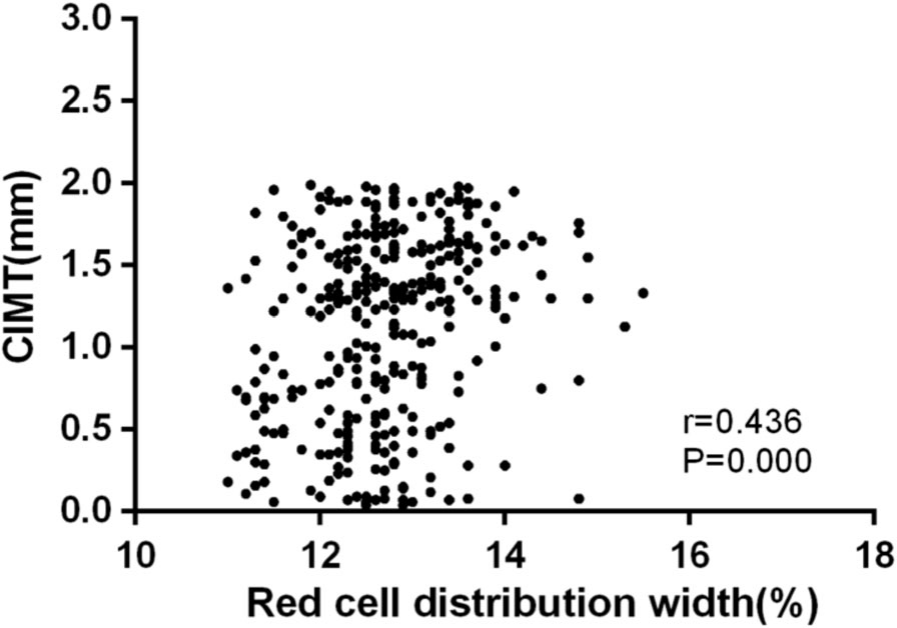
Scatter plot between RDW and CIMT

The univariate correlation analysis was conducted among CIMT and other variables. Results show CIMT is positively related to RDW (*r* = 0.436, *P* < 0.001, Fig. 1). Similarly, CIMT also has significantly positive relationship with smoking (*r* = 0.253, *P* < 0.001), drinking history (*r* = 0.209, *P* = 0.042) (Spearman’s correlation), waist circumference (*r* = 0.287, *P* < 0.001), SBP (*r* = 0.425, *P* = 0.025), triglyceride (*r* = 0.225, *P* = 0.016), LDL-C (*r* = 0.125, *P* = 0.035), serum uric acid (*r* = 0.254, *P* < 0.001), blood platelet (*r* = 0.131, *P* = 0.018) (Pearson correlation). And the CIMT was negatively associated with hemoglobin (*r* = −0.236, *P* = 0.032). The main results are shown in Table 2.

**Table 2:** Univariate correlation analysis between CIMT and other parameters in patients with MS

Variables	Correlation coefficient	*P* value
Age (year)	0.029	0.562
Sex (male, %)	0.038	0.396
Smoking, yes	0.253	0.000
Drinking history, yes	0.209	0.042
Hypertension, yes	0.065	0.635
Diabetes mellitus, yes	0.482	0.821
Body mass index, kg/m^2^	0.078	0.125
Waist circumference, cm	0.287	0.000
Waist to hip ratio	0.097	0.051
Systolic blood pressure, mmHg	0.425	0.025
Diastolic blood pressure, mmHg	0.068	0.687
Triglyceride, mmol/dL L	0.225	0.016
HDL-C, mmol/dL	−0.064	0.496
LDL-C, mmol/dL	0.125	0.035
Total cholesterol, mmol/dL	0.059	0.186
Fasting plasma glucose, mmol/dL	0.058	0.758
HbA1c (%)	0.046	0.248
Serum creatinine, mmol/dL	0.047	0.293
Serum uric acid, μmol/L	0.254	0.000
Blood urea nitrogen, (mmol/L)	0.064	0.355
Hs-CRP, mg/dL	−0.013	0.779
Red blood cell, ×10^12^/L	−0.052	0.348
White blood cell, ×10^9^/L	0.176	0.045
Red cell distribution width (%)	0.436	<0.001
Blood platelet, ×10^9^/L	0.131	0.018
Hemoglobin,g/L	−0.236	0.032

*HDL-C* high-density lipoprotein cholesterol, *LDL-C* low-density lipoprotein cholesterol, *Hs-CRP* high-sensitive C-reactive protein, *CIMT* carotid intimal-medial thickness

### Multiple regression analysis

**Fig. 2:**
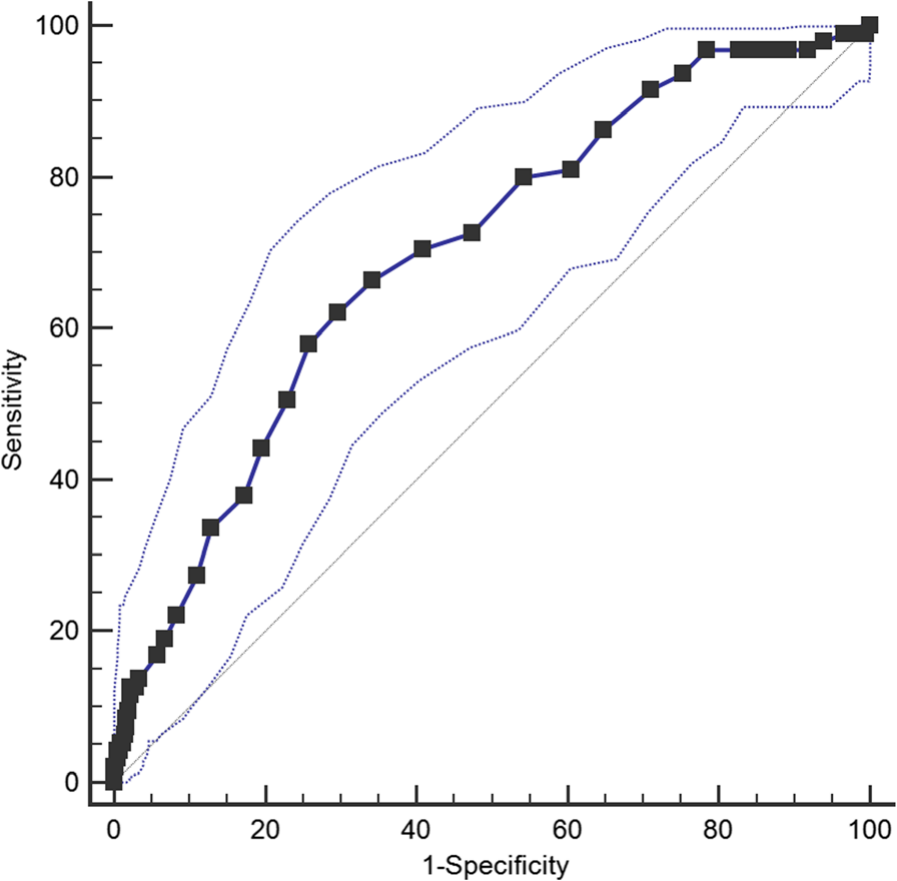
ROC curve was used to analyze the cutoff point of RDW in detecting the increased CIMT

Also multiple linear and logistic regression analysis were conducted to further explore the possible CIMT and RDW relationship. Both regression models include the following variables: age, sex, smoking, drinking history, hypertension, diabetes mellitus, BMI WC, WHR, SBP, DBP, TG, HDL-C, LDL-C, TC, FPG, HbAlc, serum creatinine, uric acid, BUN, Hs-CRP, red blood cell, white blood cell, blood platelet, RDW, and hemoglobin. The multiple linear regression reveals a positive association between RDW and CIMT (β = 0.487, *P* < 0.001), independent of potential confounding factors. Specifically, CIMT increases by about 0.5 mm with one percent change of RDW. The logistic regression indicates that RDW is a predictor of CIMT ≥ 1 mm. Compared with the first quartile, patients in third and fourth quartile have obviously higher risk of carotid artery atherosclerotic (OR = 1.41, 95% CI:1.01–197; OR = 2.10, 95% CI: 1.30–3.40). Also a receiver operator characteristic (ROC) curve was used to analyze the predictive values of RDW in detecting thickened CIMT in the Mets population. As shown in Tables 3 and 4, the cutoff value of RDW was 13.9% with a sensitivity of 62.1% and a specificity of 70.3%. The Youden index is 0.324, and the area under ROC curve is 0.698 (95% CI: 0.657–0.738, Fig. 2).

**Table 3:** Stepwise multiple linear regression analysis for the effect of independent variables on CIMT

Variable	*Beta* ^a^	S.E.	*t*	*P*
Constant	−1.822	1.151	24.09	<0.001
Smoking	0.150	4.572	4.07	0.001
Waist circumference	0.125	0.632	3.76	0.004
RDW	0.487	2.044	12.69	<0.001
Serum uric acid,	0.301	0.113	7.689	<0.001
Triglyceride	0.115	1.562	3.94	0.012
Systolic blood pressure	0.193	0.140	5.25	<0.001

^a^Standardized coefficients

**Table 4:** Multiple logistic regression for CIMT in patients with MS

Variables	*B*	S.E.	Wald	*P* value	OR (95% CI)
Smoking	0.290	0.122	5.30	0.021	1.60 (1.15–2.22)
Waist circumference	0.522	0.168	9.68	<0.001	1.69 (1.21–2.34)
Systolic blood pressure	0.663	0.275	5.83	0.016	1.94 (1.13–3.32)
Triglyceride	0.530	0.208	6.47	0.011	1.70 (1.13–2.55)
Serum uric acid	0.352	0.168	4.39	0.036	1.42 (1.02–1.98)
RDW					
The lowest quartile					Reference
The second quartile	0.047	0.182	0.07	0.797	1.05 (0.73–1.50)
The third quartile	0.346	0.170	4.16	0.041	1.41 (1.01–1.97)
The fourth quartile	0.744	0.245	9.24	0.001	2.10 (1.30–3.40)

## Discussion

Previous studies show that patients with increased CIMT have a higher risk of cardiovascular diseases than the unaffected patients [22, 23]. The elevated RDW is a potential predictor of carotid artery atherosclerosis in patients with ischemic stroke [16]. Early identification is important for such a progressive disease like carotid artery atherosclerosis, and the role of RDW could differ among people settings. The present study finds that patients with CIMT > 1 mm have higher RDW than those with CIMT ≤ 1 mm, and the elevated RDW is associated with increased CIMT, independent of a number of potential confounding factors. Our findings more strongly support the role of RDW role in the progression of carotid artery atherosclerosis.

The relationship between RDW and elevated CIMT may be attributed to two reasons. The first and more important reason could be cytokine-mediated inflammatory response. Since great efforts have been made to explore the mechanism of atherosclerosis in the past several years, it becomes clearer that inflammation is a main reason. As is well known, many inflammatory cytokines play significant roles in the progression of increased CIMT, which results in carotid artery atherosclerosis. As reported, people with increased CIMT have a higher hs-CRP level than the unaffected participants, and the levels of interleukin-6 and tumor necrosis factor-α are also increased [24, 25]. The increased CIMT is often accompanied by subclinical atherosclerosis. It is suggested that inflammatory arthropathies could be improved by anti-tumor necrosis factor-α therapy in the progression of CIMT [5]. Our study confirms that hs-CRP is indeed elevated in patients with CIMT > 1 mm. These findings indicate inflammation widely occurs in patients with elevated CIMT. More importantly, a large cohort study uncovers a strong, graded and positive relationship RDW and hs-CRP [10]. Moreover, erythropoietin concentration is related to aggravated inflammation in non-anemic adults status, and is a negatively associated in anemic population [26]. This finding proves that RDW could predict CIMT in MetS patients. Moreover, RDW in the highest quartile is associated with higher incidence of total stroke and cerebral infarction, and a close relationship between high RDW and IMT, as well as the incidence of carotid plaque, is identified in hypertensive inpatients [27, 28]. We confirm this relationship in other population settings. This one is different from the previous studies, which show an association between higher RDW and reduced eGFR indicating that lower eGFR could predict higher levels of RDW [14]. However, in the present study, patients with CIMT < 1 mm have significantly lower eGFR (72.8 ± 35.5) when compared to the case group with eGFR of 95.5 ± 21.3 ml/min/1.73 m^2^. This can be explained below. First, previous studies are limited to the hypertension or diabetic population, but the present study has normal renal function. There are differences in life styles such as smoking and drinking. The control group seems to have higher rate of smoking and drinking. The elevated CIMT is a common symptom in the elderly. CIMT consistently increases with age and even tend to affect males [29] , while age and gender could both influence RDW [30]. This point further proves the strong relationship between RDW and CIMT. Previous studies tend to attribute these relationships to inflammatory responses, but there is still no accurate and elaborated mechanism. Thus, further investigation is required.

Nevertheless, this study has several limitations. First, it is based on a cross- sectional design, which limits the causal relationship between RDW and CIMT. However, we believe the relationship is probably reasonable after the summarization of previous findings. Second, only MetS patients were included, so the findings should be applied to other people settings with caution. Third, some people had been diagnosed with hypertension or mellitus diabetes, who might be receiving drug treatment. We were inaccessible to this information due to some reasons, and we did not include drug history in the final analysis, which could have some influences on the final results. Finally, we confirmed the possible relationship between RDW and elevated CIMT, but we did not illustrate the accurate mechanism. Thus, further investigations are needed.

## Conclusions

In conclusion, high RDW is related to the increased CIMT in MetS patients, which highlights the role of RDW in the progression of elevated CIMT in MetS patients. As a convenient and inexpensive examination, RDW combined with other clinical examinations could be an effective index for detection of early early carotid artery atherosclerosis. Future studies should focus on the exact mechanism.

## Abbreviations

BMI: Body mass index
BUN: Blood urea nitrogen
CI: Confidence interval
CIMT: Carotid intima-media thickness
DBP: Diastolic pressure
eGFR: Estimated glomerular filtration rate
FPG: Fast plasma glucose
HDL-C: High density lipoprotein-cholesterol
Hs-CRP: High-sensitivity C-reactive protein
LDL-C: Low density lipoprotein-cholesterol
MetS: Metabolic syndrome
OR: Odds ratio
RDW: Red cell blood distribution width
SBP: Systolic blood pressure
TC: Total cholesterol
WHR: Waist to hip ratio
